# Genetic parameters for periodontal disease: an analysis of electronic dental treatment records linked to pedigree, genomic, sociodemographic and clinical data

**DOI:** 10.23889/ijpds.v1i1.373

**Published:** 2017-04-19

**Authors:** Mairead Bermingham, Archie Campbell, David Porteous, Angus Walls

**Affiliations:** 1 Centre for Genomics and Experimental Medicine, Institute of Genetics and Molecular Medicine, University of Edinburgh; 2 Edinburgh Dental Institute, University of Edinburgh

## Background

Electronic health records provides unprecedented opportunity for their re-use in genetic epidemiological research. However, electronic health records data from clinical settings, such as dental practices may be inaccurate or of insufficient granularity to be of use in research. In this study, we wish to determine the utility of National Health Service (NHS) electronic dental treatment records in genetic epidemiological research.

## Objective

To estimate the heritability of periodontal disease using NHS electronic dental treatment records linked to health and non-health data within the Generation Scotland: Scottish Family Health Study (GS:SFHS).

## Approach

We linked 852,355 NHS Scotland electronic dental treatment records from April 2000 to July 2015 to 20,626 participants within the GS:SFHS with pedigree, genomic, sociodemographic and clinical data. We then conducted a proof-of-principle genetic epidemiological analysis using periodontal (gum) disease treatment records. The data set analysed, consisted of 160,508 dental treatment records from 13,717 study participants; 3,387 of which were periodontal treatment records (from 2,192 study participants). We adjusted for the effects of previous treatment record, interval since last treatment, age, sex, treatment year, and treatment month, Scottish index of multiple deprivation, alcohol consumption, diabetes diagnosis, and smoking status in a linear model in the statistical software ASReml. We then calculated the mean risk of periodontal disease for each study participant based on residuals extracted from the aforementioned model. Genome-complex trait analysis (GCTA; with correction for population stratification) was used to estimate the pedigree and genomic based heritability of periodontal disease.

## Results

We estimate the familial heritability of periodontal (gum) disease at 10.42% (95% confidence interval 5.97-14.88%). The genomic component did not contribute significantly to the heritability estimate.

## Conclusion

we have demonstrated the usefulness of electronic dental treatment records in population based genetic epidemiological research .This study has also, to the best of our knowledge provided the first population based estimates of the genetic parameters for periodontal disease; confirming its familial nature. This invaluable and unique data resource will allow the acceleration of oral health research in Scotland and the exploration of research questions that could not be considered previously.

